# MiR-760 targets HBEGF to control cartilage extracellular matrix degradation in osteoarthritis

**DOI:** 10.1186/s13018-023-03664-1

**Published:** 2023-03-10

**Authors:** Yingchun Zhu, Chi Zhang, Bo Jiang, Qirong Dong

**Affiliations:** 1 Department of Orthopaedic Surgery, The Second Affiliated Hospital of Soochow University, Soochow, 215000 China; 2 Department of Orthopedic Surgery, Ningbo First Hospital, No. 59, LiuTing Street, Ningbo, 315010 China

**Keywords:** Osteoarthritis, Chondrocytes, miR-760, HBEGF, Matrix homeostasis

## Abstract

The present study was developed to explore whether microRNA (miR)-760 targets heparin-binding EGF-like growth factor (HBEGF) to control cartilage extracellular matrix degradation in osteoarthritis. Both miR-760 and HBEGF expression levels were analysed in human degenerative cartilage tissues and in interleukin (IL)-1β/tumour necrosis factor (TNF)-α-treated chondrocytes in vitro. A series of knockdown and overexpression assays were then used to gauge the functional importance of miR-760 and HBEGF in OA, with qPCR and western immunoblotting analyses. Bioinformatics assays were used to identify putative miR-760 target genes, with these predictions then being validated through RNA pulldown and luciferase reporter assays. A murine anterior cruciate ligament transection model of OA was then established to prove the in vivo relevance of these findings. These experiments revealed that human degenerative cartilage tissues exhibited significant increases in miR-760 expression with a concomitant drop in HBEGF levels. IL-1β/TNF-α-treated chondrocytes also exhibited significant increases in miR-760 expression with a concomitant drop in HBEGF expression. When chondrocytes were transfected with either miR-760 inhibitor or HBEGF overexpression constructs, this was sufficient to interfere with degradation of the extracellular matrix (ECM). Moreover, miR-760 was confirmed to control chondrocyte matrix homeostasis by targeting HBEGF, and the overexpression of HBEGF partially reversed the effects of miR-760 mimic treatment on the degradation of the cartilage ECM. When OA model mice were administered an intra-articular knee injection of an adenoviral vector encoding a miR-760 mimic construct, cartilage ECM degradation was aggravated. Conversely, the overexpression of HBEGF in OA model mice partially reversed the effects of miR-760 overexpression, restoring appropriate ECM homeostasis. In summary, these data indicated that the miR-760/HBEGF axis plays a central role in orchestrating the pathogenesis of OA, making it a candidate target for therapeutic efforts in OA.

**Supplementary Information:**

The online version contains supplementary material available at 10.1186/s13018-023-03664-1.

## Introduction

Osteoarthritis (OA) is the second most common cause of musculoskeletal disability and a prominent driver of joint pain in affected patients [1]. At present, therapeutic strategies used to treat OA primarily focus on relieving symptoms through a combination of surgery, injections, and/or physical therapy. However, these approaches cannot restore normal joint function or prevent further disease progression in treated patients [2]. Moreover, the mechanisms driving the onset and pathological progression of OA are limited, hampering efforts to develop novel therapeutic interventions.

Prior research efforts have largely explored the biomechanical and morphological alterations exhibited by OA patients, as these changes, together with cellular metabolic dysregulation-induced nutritional dysfunction, are thought to contribute to eventual joint degeneration [3, 4]. However, more recent work has suggested that altered cellular functionality and cell death within joints may be a more critical driver of OA pathogenesis [5]. Under physiological conditions, chondrocytes maintain appropriate joint homeostasis by secreting extracellular matrix (ECM) components and a range of other regulatory mediators. As such, chondrocyte function is a key determinant of joint function [6, 7]. Inflammation is a hallmark of the pathological progression of OA and a range of other degenerative diseases, with interleukin (IL)-1β, tumour necrosis factor (TNF)-α and other inflammatory cytokines functioning to promote altered ECM metabolism and the senescence or apoptosis of target chondrocytes [8, 9]. As such, strategies aimed at modulating chondrocyte ECM metabolic activity may be an effective means of treating OA.

MicroRNAs (miRNAs) are 20–24 nucleotide transcripts that can control diverse processes including proliferation, differentiation, and immune response induction through the post-transcriptional regulation of specific target mRNAs [10–13]. Several miRNAs have been found to play important regulatory roles in OA [14, 15]. For example, miR-335-5p has been shown to activate autophagic activity, thereby mitigating chondrocyte inflammation and slowing OA disease progression [16], while miR-1271 can influence the ECM metabolizing activity of chondrocytes to alter OA incidence and severity [17]. As such, miRNAs are important regulators of joint homeostasis that warrant further study in an effort to highlight novel therapeutic targets and to gain additional insight into the regulatory pathways that shape the progression of OA.

MiR-760 was recently reported to be involved in the development of inflammation. MiR-760-3p could regulate Map3k8 to activate the NF-κB pathway in cerebral ischemia [18], while the ceRNA network circNTRK2/miR-760/LAT was dysregulated in obese patients, which was also associated with inflammation [19]. In addition, miR-760 was found to target Myo18b in the context of rheumatoid arthritis to control skeletal muscle cell proliferation [20]. Osteoarthritis is the most common arthritic disease, and whether miR-760 plays a similar regulatory role in OA has yet to be established. Accordingly, the present study was developed to assess the expression and function of miR-760 in human degenerative cartilage tissues and IL-1β/TNF-α-treated chondrocytes to gain insight into its regulatory role in the context of abnormal chondrocyte-regulated matrix homeostasis. Through these experiments, the miR-760/heparin-binding EGF-like growth factor (HBEGF) axis was identified as a novel regulator of the progression of OA, underscoring its potential utility as a target for therapeutic intervention in patients affected by this debilitating disease.

## Materials and methods

### Ethics statement

The Ethical Committee of the Ningbo City First Hospital approved all human studies, which were conducted in accordance with the Declaration of Helsinki (Approval Number: 2021-R142). Informed consent was obtained before experimentation with human subjects. All animal studies were approved by the Animal Care and Use Committee of Ningbo University and were performed as per the NIH Guide for the Care and Use of Laboratory Animals (Approval Number: 11430).

### Human tissue collection and cell culture

**Fig. 1:**
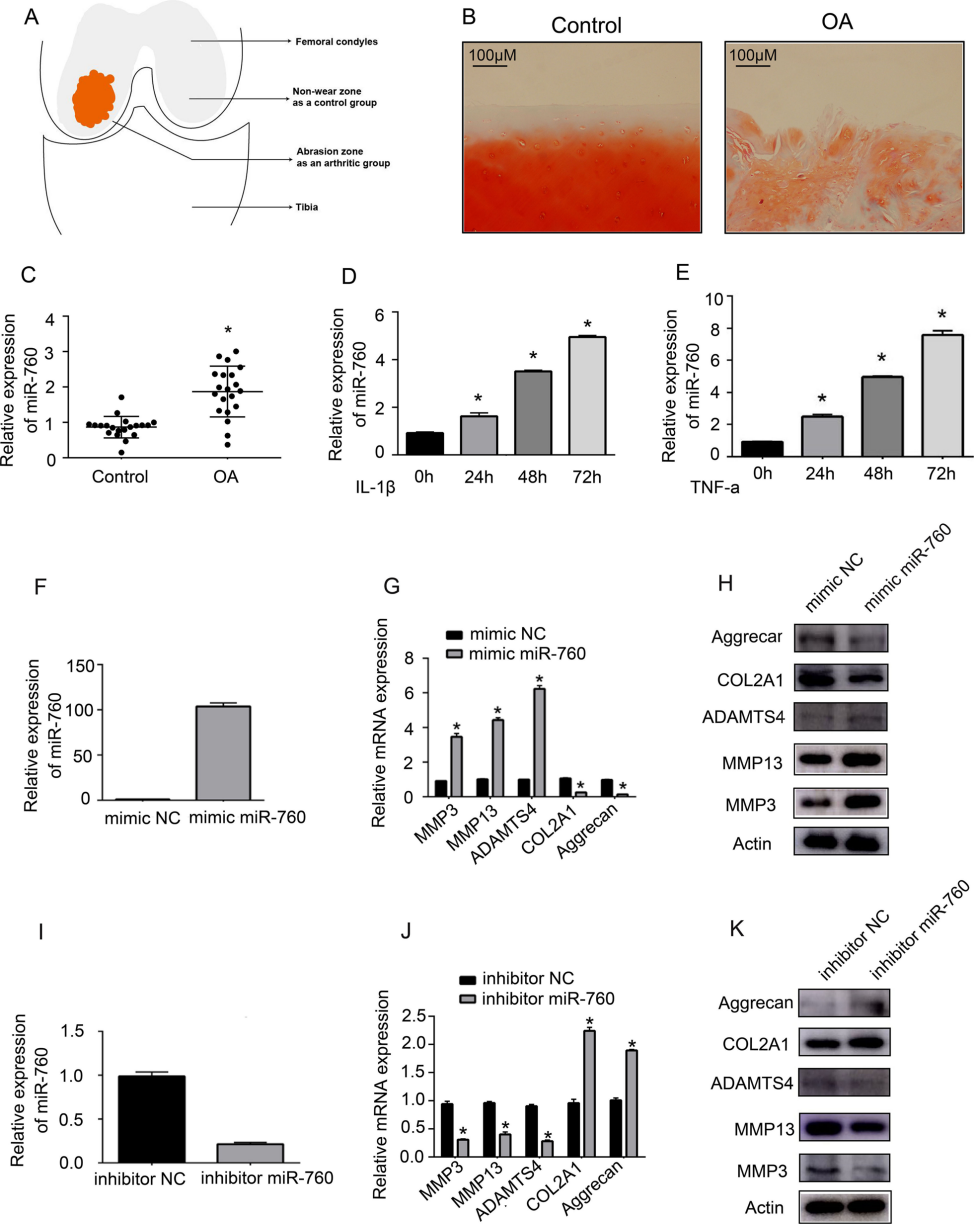
Degenerative OA-associated cartilage exhibited higher levels of miR-760 expression than nondegenerative cartilage. **A** Schematic overview of the classification of degenerative and nondegenerative cartilage tissue samples. **B** Representative safranin-O/fast green staining of cartilage from control or OA patients. Scale bar: 100 µm. **C** MiR-760 expression levels in OA and non-OA cartilage samples. *n* = 20 (twenty different donors). Student's t test (t test) was used for comparisons between two groups. *p < 0.05. **D**, **E** The expression of miR-760 in chondrocytes stimulated with TNF-α (10 ng/mL) and IL-1β (10 ng/mL) for 24 h, 48 h, and 72 h. *n* = 3 (1 technical replicate on 3 different donors). Data were analysed by one-way analysis of variance (ANOVA) followed by Tukey’s post hoc test for comparison between the control and treatment groups. *, *p* < 0.05 compared with 0 h. MiR-760 regulates chondrocyte-mediated degradation of the extracellular matrix. **F**, **I** After 24 h of transfection, the efficiency of the overexpression or knockdown of miR-760 in chondrocytes transfected with the indicated constructs. *n* = 3 with 3 technical replicates. Student's t test (t test) was used for comparisons between two groups. **G**, **J** After 24 h, cartilage metabolism-related gene expression was analysed in chondrocytes via qPCR following the overexpression or inhibition of miR-760. *n* = 3 with 3 technical replicates. Student's t test (t test) was used for comparisons between two groups. *p < 0.05. **H**, **K** After 48 h, cartilage metabolism-related protein expression was examined in chondrocytes following the overexpression or inhibition of miR-760 via Western immunoblotting. *n* = 3; three different donors. miRNAs, microRNAs; qPCR, real-time quantitative PCR. OA, osteoarthritis; TNF, tumour necrosis factor; IL, interleukin

Human tissue and cell culture samples were harvested from 20 end-stage symptomatic OA patients undergoing total knee joint replacement surgery. A schematic representing the target locations for the collection of degenerative cartilage samples from internal worn areas and nondegenerative samples from external nonabraded areas is shown in Fig. 1A. Twenty paired clinical OA and control tissues were used for miR-760 and HEBGF expression analysis. Nondegenerative samples from three different donors were used for cell extraction and culture. Human chondrocytes were prepared by cutting isolated samples of articular cartilage into pieces and digesting them for 6 h in DMEM containing collagenase II (2 mg/mL) in a 37 °C incubator. Supernatants were then passed through a 0.075 mm filter, and the filtrate was then centrifuged. Then, the bottom sediment was washed two times using PBS and cultured in DMEM containing 10% fetal bovine serum (FBS; Thermo Fisher Scientific, MA, USA) and 1% penicillin‒streptomycin in a 37 °C humidified 5% CO_2_ incubator. Human chondrocytes were collected from 3 patients and used for in vitro experiments.

### Cell treatment

Primary human articular chondrocytes were treated with TNF-α (10 ng/mL) and IL-1β (10 ng/mL) (Sigma-Aldrich) when 70–80% confluent, and total RNA was then harvested from these cells after 0, 24, 48, or 72 h to assess inflammation-induced changes in miR-760 and HBEGF levels.

### Transfection

Human chondrocytes were added to 6-well plates and incubated to 70% confluence, after which they were transfected with miR-760 mimic/inhibitor, overexpression HBEGF (OE HBEGF), knockdown HBEGF (shHBEGF), or corresponding negative control constructs (Ruibo, Guangzhou, China) using Lipofectamine 3000 (Invitrogen, CA, USA). At 24 h and 48 h post-transfection, cells were harvested for downstream use. Transfection efficiency was assessed through qPCR and Western immunoblotting. The above constructs sequences were as follows:mimic miR-760 sequence: 5'-CGGCUCUGGGUCUGUGGGGA-3', 5'-UCCCCACAGACCCAGAGCCG-3'; negative control of mimic miR-760 sequence: 5'-UUCUCCGAACGUGUCACGUTT-3', 5'-ACGUGACACGUUCGGAGAATT-3'; inhibitor miR-760 sequence: 5'-UCCCCACAGACCCAGAGCCG-3'; negative control of inhibitor miR-760 sequence: 5'-CAGUACUUUUGUGUAGUACAA-3'. In addition, the pLVX vector was used for HBEGF overexpression and knockdown. Vector sequence diagrams are described in Additional file 1.

### RNA extraction and quantitative real-time PCR analysis

In this study, mRNA was isolated from cultured cells and knee articular cartilage tissues. The cultured cells were rinsed with PBS and lysed in RNA-Solv^®^ Reagent (Omega Bio-tek, Norcross, GA, USA). The knee cartilage samples were placed in paired RNase-Free 1.5 EP tubes with four ground beads (5 mm in diameter) and frozen with liquid nitrogen. Subsequently, the tissues were pulverized and homogenized using Tissuelyser-24 (Jingxin, Shanghai, China). The TissueLyser was operated twice for 30 s at 45 Hz. The above tissue powder (50–100 mg) was lysed in Omega RNA-Solv^®^ Reagent and RNA was isolated using the E.Z.N.A.^®^ Total RNA Kit I (Omega Bio-tek) according to manufacturer’s protocol. MiRNA levels were extracted using a miRNA Isolation Kit (Ambion). RNA was stored at − 80 °C. Reverse transcription was performed using 1.0 µg total RNA and then used to prepare cDNA using miRNA and HiFiScript cDNA kits (CWBIO, Beijing, China), which were used to investigate the expression of miRNA and mRNA, respectively. All qPCRs were performed in a 20 µL volume using appropriate primers (1 µL; Sangon Biotech, Shanghai, China), cDNA (1 µL), and a ROX-containing UltraSYBR Mixture (CWBIO) with an ABI 7500 Sequencing Detection instrument (Applied Biosystems, CA, USA). The thermocycler settings were as follows: 40 cycles of 95 °C for 5 s and 60 °C for 24 s. U6 was used as an internal control for microRNA, whereas *β*-actin served as the control for messenger RNA. The cycle threshold (Ct) values were collected and normalized to the level of U6 or β-actin, with three samples per group. The relative mRNA level of each target gene was calculated by using the 2^−ΔΔCt^ method. Primer sequences are shown in Table 1.

**Table 1:** Primer sequences for qPCR

Gene	Primers
MiR-760	Forward: UUCUCCGAACGUGUCACGUTT Reverse: ACGUGACACGUUCGGAGAATT
MMP3	Forward: AGTCTTCCAATCCTACTGTTGCT Reverse: TCCCCGTCACCTCCAATCC
MMP13	Forward: ACTGAGAGGCTCCGAGAAATG Reverse: GAACCCCGCATCTTGGCTT
ADAMTS4	Forward: GAGGAGGAGATCGTGTTTCCA Reverse: CCAGCTCTAGTAGCAGCGTC
COL2A1	Forward: TGGACGATCAGGCGAAACC Reverse: GCTGCGGATGCTCTCAATCT
Aggrecan	Forward: ACTCTGGGTTTTCGTGACTCT Reverse: ACACTCAGCGAGTTGTCATGG
HBEGF	Forward: ATCGTGGGGCTTCTCATGTTT Reverse: TTAGTCATGCCCAACTTCACTTT
CBL	Forward: TGGTGCGGTTGTGTCAGAAC Reverse: GGTAGGTATCTGGTAGCAGGTC
CAMK2G	Forward: ACCCGTTTCACCGACGACTA Reverse: CTCCTGCGTGGAGGTTTTCTT
MAP2K1	Forward: CAATGGCGGTGTGGTGTTC Reverse: GATTGCGGGTTTGATCTCCAG
ADCY1	Forward: AGGCACGACAATGTGAGCATC Reverse: TTCATCGAACTTGCCGAAGAG
RPS6KA3	Forward: CGCTGAGAATGGACAGCAAAT Reverse: TCCAAATGATCCCTGCCCTAAT
U6	Forward: CTCGCTTCGGCAGCACA Reverse: AACGCTTCACGAATTTGCGT
β-actin	Forward: AGATGTGGATCAGCAAGCAG Reverse: GCGCAAGTTAGGTTTTGTCA

### Western immunoblotting

Expression of proteins of interest in chondrocytes and articular cartilage tissues were explored through western blot analysis. Briefly, cultured chondrocytes and previously ground tissues powder (100 mg) were lysed for 1 h using ice cold 1 × RIPA lysis buffer (Beyotime) supplemented with phenylmethanesulfonyl fluoride (PMSF, 1 mM, Beyotime). The lysates were centrifugated for 10 min at 4 °C and 12,000 rpm. The supernatant was transferred to a new tube for protein quantification. A standard curve was prepared with gradient concentration of BSA according to the instructions of a BCA quantification kit (Beyotime, China). For western blotting assay, the protein samples were mixed with 5 × loading buffer and heated for 10 min at 98 °C. Thereafter, 30 ug protein samples were separated by 10%–12.5% SDS-PAGE gels and transferred to polyvinylidene fluoride membranes (Bio-Rad, USA). The membrane was rinsed with TBS-T, following with 5% nonfat milk for 1.5 h. Then, the membranes were incubated with primary antibodies specific to HBEGF (1:200, Abcam, Cat# ab92620), MMP3 (1:1000, Abcam, Cat# ab137659), MMP13 (1:1000, Abcam, Cat# ab84594), ADAMTS4 (1:1000, Abcam, Cat# ab84792), COL2A1 (1:1000, Abcam, Cat# ab34712), Aggrecan (1:100, ABclonal, Cat# A8536), or β-actin (1:2000) (Cell Signaling Technology, Cat# 4970) at 4 °C overnight. The blots were then rinsed and probed with secondary HRP-conjugated anti-rabbit or anti-mouse antibodies (Beyotime Institute of Biotechnology, Nantong, China), and proteins were detected using an ECL kit (Santa Cruz Biotechnology, TX, USA).

### Predictive bioinformatics analyses

Two databases were used to predict miR-760 target genes and downstream signalling pathways, miRDIP (http://ophid.utoronto.ca/miRDIP/index.jsp) and DAVID (DAVID Functional Annotation Bioinformatics Microarray Analysis (ncifcrf.gov)) [21]. Briefly, the potential targets of miRNAs were predicted with the miRDIP database, and the genes with a high minimum score (top 5%) were selected as the targets of the common miRNAs. Subsequently, the potential targets of differentially expressed miRNAs were used for KEGG enrichment analysis, and the genes were annotated and analysed by the DAVID database.

### Luciferase reporter assays

HEK293T cells were cotransfected with miR-760 mimic or control constructs together with 3ʹ-UTR-Luc reporter plasmids containing wild-type (WT) or mutated miR-760 binding sites from the HBEGF 3'-UTR using Lipofectamine 3000 (Invitrogen). For mutant HBEGF reporter constructs, we used the previously predicted binding site sequence of miR-760 and HBEGF (http://ophid.utoronto.ca/mirDIP/index.jsp#r). Then, we altered the gene at the binding site and transferred the sequence into an overexpression vector to form a mutant of the gene (detailed sequences are shown in Supplemental Materials 2). A Luciferase Reporter Gene system (Sigma‒Aldrich, MO, USA) was then used based on the provided directions to detect luciferase activity.

### Adenovirus preparation

To determine whether miR-760 or HBEGF plays a role in OA progression in vivo, adeno-associated virus (AAV, virus titer 1.3 × 10^12^vg/mL, catalogue number: GC20200717HZCY-AAV01) was obtained from Hanheng (Hanheng Biotechnology Co., Ltd., Shanghai, China) to overexpress miR-760 or HBEGF in the OA mouse model. The vector sequence diagram is described in Additional file 1.

### Experimental animal model

Referring to the common experimental animal randomization method and the 3R principle, 36 C57BL/6 male mice (6 weeks old) were randomized into the following three groups: a sham surgery group (incision of the right knee, lack of ACL transection surgery) and two groups in which the right knee articular cartilage underwent anterior cruciate ligament transection (ACLT) to establish an animal model of OA (*n* = 12). The two OA experimental groups were administered intra-articular injections of either AAV-miR-760 mimic or AAV-miR-760 mimic + AAV-OE HBEGF immediately after ACLT surgery (0 weeks). The injection procedure was repeated after 4 weeks, and the mice were sacrificed at 4 weeks after the second injection. The right knee joints of each group were harvested for the extraction of RNA (*n* = 3) and proteins (*n* = 3), with OA progression being assessed based on the expression of HBEGF, MMP3, MMP13, ADAMTS4, COL2A1, and Aggrecan. Briefly, mice were sacrificed with the nape facing up. Then, the front legs were immobilized and the skin and soft tissue were removed on the hind leg to make an incision at the knee joint. After exposing the tibial plateau, the surface resembling a regular translucent sphere (articular cartilage) was severed and processed for RNA and protein studies. In addition, the right knee joints of the remaining mice (*n* = 6) were dissected and processed for safranin-O/fast green staining and immunohistochemistry staining.

### Histological analysis and OARSI score

After surgery, mice that received different treatments were kept separately, and each treatment group had two cages (3 mice in a 400 square inch cage). At 8 weeks post-surgery, cartilage specimens were fixed in 4% paraformaldehyde for paraffin embedding. Each paraffin-embedded cartilage sample was sectioned at 5 μm, and every tenth section was stained with 0.1% safranin O solution and 0.001% Fast Green solution (Sigma‒Aldrich, St. Louis, MO, USA). For simple histologic scoring of OA in the mouse, we used an approved 0–6 subjective scoring system [22]. Histologic scores were evaluated in a blinded manner according to a grading scale (0 for normal cartilage, 0.5–4 for moderately degenerated cartilage, and 5–6 for severely degenerated cartilage).

### IHC staining

The paraffin-embedded tissue sections were deparaffinized and rehydrated following standard procedures. Sections were incubated with 3% H_2_O_2_ to block endogenous peroxidase activity and antigen retrieval was performed in citrated buffer at 110 ℃, for 5 min in a pressure cooker. After the citrated buffer reached room temperature, the sections were removed and incubated overnight with the primary antibodies COL2A1 (1:200, bioss, bs-10589R) and SOX9 (1:1000, Abcam, Cat# ab185966) at 4 ℃, followed by incubation with an HRP conjugated secondary antibody (Beyotime Institute of Biotechnology, Inc., Nantong, China) for 2 h at room temperature. Peroxidase binding for both COL2A1 and SOX9 was visualized using diaminobenzidine. Then, the nuclei were counterstained with hematoxylin, while the slides were dehydrated, mounted, and analyzed with a light microscope. For the quantitative analysis, all positively stained cells, including those in the femoral condyle and tibial plateau area, on the articular surface per specimen were counted, and the percentage of positive cells was calculated using Image-Pro Plus 6.0.

### Statistical analysis

SPSS 22.0 (SPSS, IL, USA) was used to analyse data, which are given as the means ± SEMs. Data were compared between groups using independent samples t tests or one-way ANOVAs with Tukey’s post hoc test as appropriate. *P* < 0.05 was the significance threshold.

## Results

### MiR-760 expression is relatively high in human OA tissues

Initially, degenerative and nondegenerative joint tissue samples were harvested from 20 OA patients for analysis (Fig. 1A). Safranin O and fast green staining (Fig. 1B) showed degenerative cartilage in OA-affected parts of the cartilage. Subsequent qPCR assays performed using these samples revealed significant increases in miR-760 expression in degenerative cartilage relative to nondegenerative cartilage (Fig. 1C), suggesting that this miRNA may play a role in the progression of OA.

### IL-1β and TNF-α treatment of chondrocytes promotes the time-dependent upregulation of miR-760

Inflammation is an important driver of the pathogenesis of OA. To model the relationship between such inflammation and miR-760 expression dynamics, we harvested primary human chondrocytes from nondegenerative cartilage samples and stimulated them with IL-1β/TNF-α. Subsequent qPCR analyses revealed that treatment with these inflammatory cytokines drove the time-dependent upregulation of miR-760 in these chondrocytes (Fig. 1D and E). These data were in line with the above data derived from human tissue samples.

### MiR-760 regulates chondrocyte-mediated degradation of the extracellular matrix

To gain insight into the functional role of miR-760 in the context of OA pathogenesis, we next transfected chondrocytes transfected with miR-760 mimic or inhibitor constructs, and the overexpression and knockdown efficiency was confirmed by qPCR (Fig. 1F and I). Next, the expression of anabolic enzymes associated with ECM synthesis (COL2A1, Aggrecan) and catabolic enzymes associated with ECM degradation (ADAMTS4, MMP-3, MMP-13) was assessed in these cells at the mRNA (Fig. 1G and J) and protein levels (Fig. 1H and K). MiR-760 inhibitor transfection significantly enhanced COL2A1 and aggrecan expression while suppressing MMP-3, MMP-13, and ADAMTS4 expression in these chondrocytes, and miR-760 mimic transfection yielded the opposite phenotype. As such, miR-760 may serve as a negative regulator of OA development.

### HBEGF serves as a miR-760 target gene in chondrocytes

**Fig. 2:**
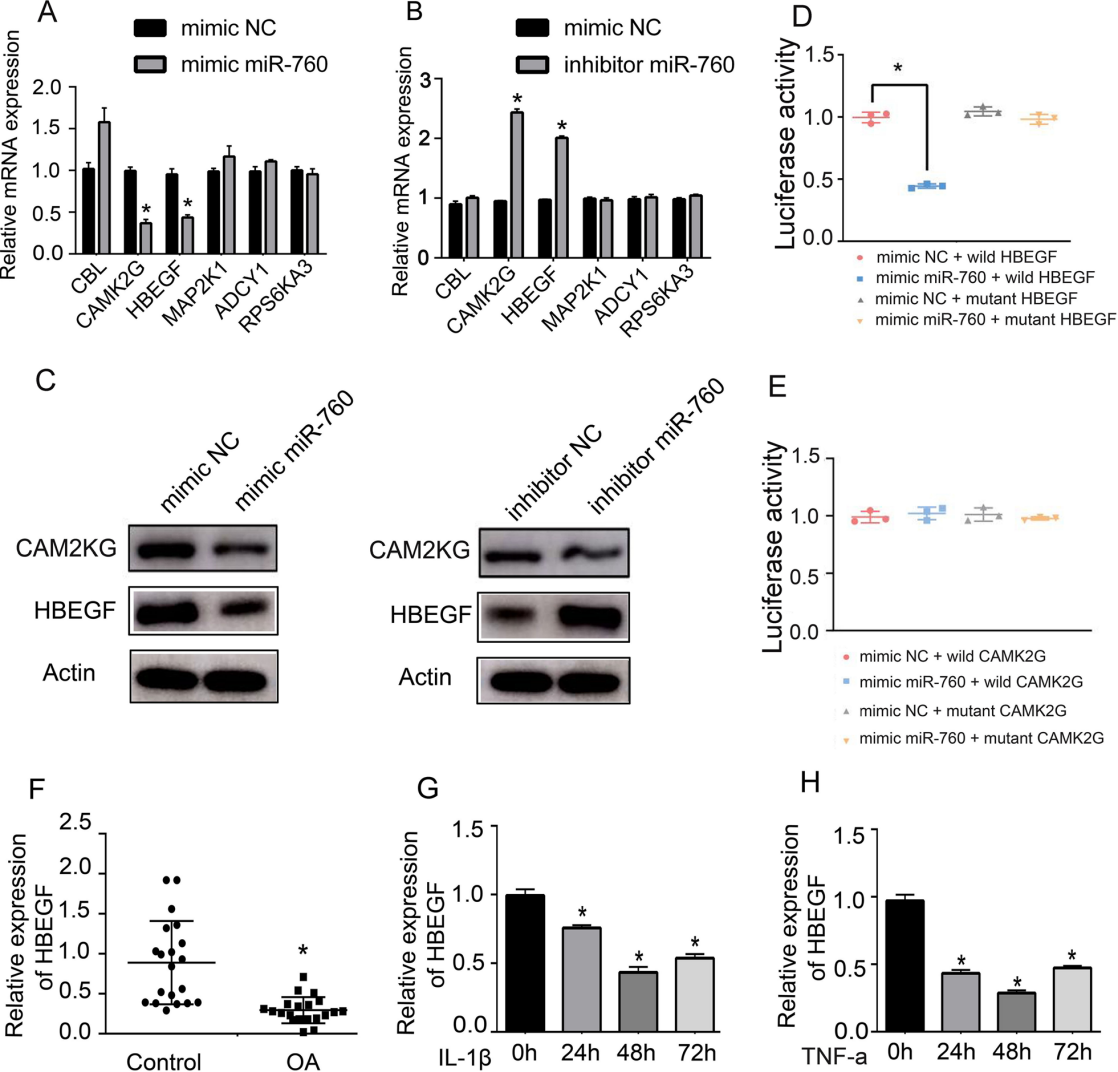
HBEGF serves as a direct target of miR-760 and mediates the function of miR-760 in chondrocytes. **A**, **B** After 24 h of transfection, the expression of 6 different putative miR-760 target genes in chondrocytes was assessed via qPCR following miR-760 overexpression or inhibition. *n* = 3 (1 technical replicate on 3 different donors). Student's t test (t test) was used for comparisons between two groups. *p < 0.05. **C** After 48 h, CAM2KG and HBEGF protein levels were analysed via western immunoblotting following miR-760 inhibition or overexpression. *n* = 3 (1 technical replicate on 3 different donors). **D**, **E** A dual-luciferase reporter assay was used to analyse interactions between miR-760 and HBEGF (or CAM2KG) mRNA. HEK-293 T cells were cotransfected with miR-760 mimic or NC and a luciferase reporter construct containing WT or MUT HBEGF (or WT or MUT CAM2KG). *n* = 3 with 2 technical replicates. Data were analysed by one-way analysis of variance (ANOVA) followed by Tukey’s post hoc test for comparison between the control and treatment groups. *p < 0.05. **F** Analyses of HBEGF expression in OA-associated degenerative and nondegenerative cartilage tissue samples. *n* = 20. Student's t test (t test) was used for comparisons between two groups. *p < 0.05. **G**, **H** HBEGF expression levels in TNF-α (10 ng/mL)- and IL-1β (10 ng/mL)-treated chondrocytes. *n* = 3 (1 technical replicate on 3 different donors). Data were analysed by one-way analysis of variance (ANOVA) followed by Tukey’s post hoc test for comparison between the control and treatment groups. *, *p* < 0.05 compared with 0 h. miRNAs, microRNAs; qPCR, real-time quantitative PCR; WT, wild type; MUT, mutant; OA, osteoarthritis; TNF, tumour necrosis factor; IL, interleukin; NC, negative control

To gain further insight regarding the mechanisms whereby miR-760 shapes the pathogenesis of OA, we used the miRDIP (http://ophid.utoronto.ca/miRDIP/index.jsp) and DAVID (DAVID Functional Annotation Bioinformatics Microarray Analysis (ncifcrf.gov)) databases to predict possible miR-760 target genes. First, we found 188 downstream genes that are highly bound to miR-760 and 11 signalling pathways associated with these 188 genes. Detailed information is displayed in Additional file 2. Second, we selected the three signalling pathways with the highest correlation (low *P* value), namely, long-term potentiation, the ErbB signalling pathway and the GnRH signalling pathway, leading to the identification of 6 possible targets. To investigate the correlation between miR-760 and these six genes, we transfected mimic miR-760 or inhibitor miR-760 into chondrocytes. After 24 h, chondrocyte RNA was extracted to detect the mRNA expression of the CBL, CAMK2G, HBEGF, MAP2K1, RPS6KA3, and ADCY1 genes. The results showed that after overexpression of miR-760 in chondrocytes, the expression of CAMK2G and HBEGF was significantly reduced, whereas knockdown of miR-760 resulted in the opposite phenotype (Fig. 2A and B). As shown in Fig. 2C, the CAM2KG and HBEGF protein expression was significantly reduced after overexpression of miR-760 in chondrocytes, while miR-760 inhibitor transfection only significantly enhanced HBEGF protein expression. A luciferase assay was then used to confirm the ability of miR-760 to bind the predicted HBEGF (or CAM2KG) 3'-UTR sequence by transfecting cells with miR-760 mimic or control constructs together with WT or mutant HBEGF (or CAM2KG) reporter constructs. Significantly reduced luciferase activity was observed following WT reporter and miR-760 mimic cotransfection, while no corresponding reduction was observed for the mutated reporter (Fig. 2D). As shown in Fig. 2E, no significant changes in luciferase activity were observed in both WT or mutant CAM2KG reporter and miR-760 mimic cotransfection. Hence, we inferred that miR-760 is most likely to directly target HBEGF and mediate the function of HBEGF in OA. As such, HBEGF was selected as a validated miR-760 target gene for further study.

### HBEGF is downregulated in OA patient cartilage tissue samples and IL-1β/TNF-α-treated chondrocytes

To assess the potential link between HBEGF and OA progression, we assessed its expression using human samples and primary chondrocytes as described above. In qPCR analyses, significant reductions in HBEGF expression were observed in degenerative cartilage samples from OA patients relative to nondegenerative samples (Fig. 2F). Similarly, time-dependent HBEGF downregulation was observed in primary human chondrocytes treated with IL-1β/TNF-α (Fig. 2G and H).

### HBEGF controls the ability of chondrocytes to regulate ECM homeostasis

**Fig. 3:**
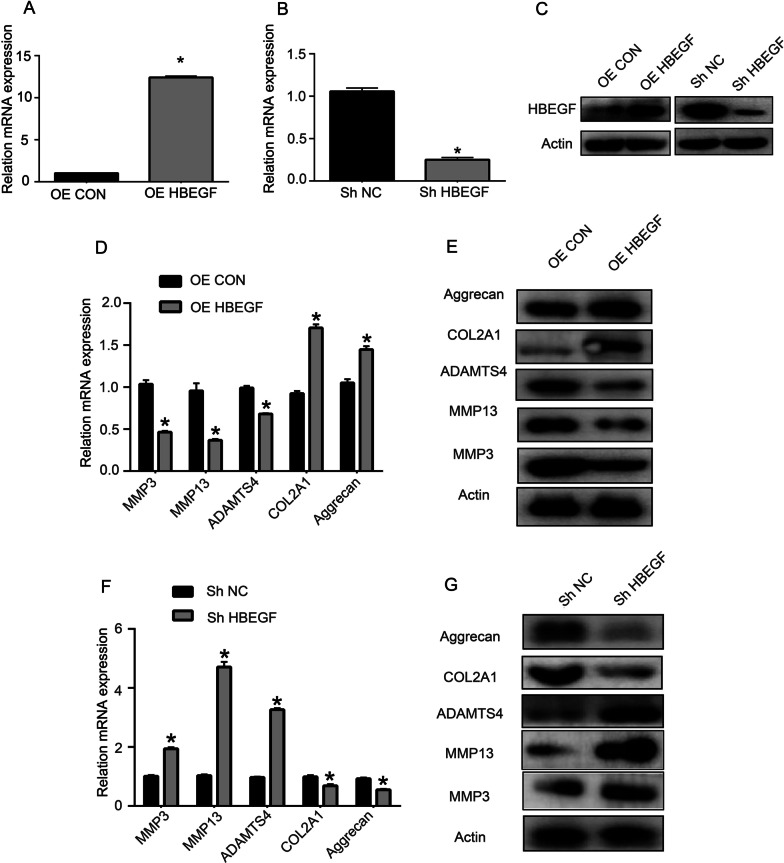
HBEGF controls the homeostasis of chondrocyte extracellular matrix. **A**–**C** After 24 and 48 h of transfection, HBEGF overexpression and knockdown efficiency at the mRNA level (A-B, *n* = 3 with 3 technical replicates, Student's t test (t test) was used for comparison between two groups. *p < 0.05) and protein levels (C, *n* = 3; three different donors) were assessed in transfected chondrocytes. **D**, **E** Chondrogenesis-related gene expression at the mRNA level (D, *n* = 3 with 3 technical replicates; Student's t test was used for comparisons between two groups. *p < 0.05) and protein levels (E, *n* = 3; three different donors) were assessed in chondrocytes following HBEGF overexpression. **F**, **G** Chondrogenesis-related gene expression at the mRNA level (F, *n* = 3 with 3 technical replicates; Student's t test was used for comparisons between two groups. *p < 0.05) and protein levels (G, *n* = 3; three different donors) were assessed in chondrocytes following HBEGF knockdown. OE, overexpression; Sh, short hairpin RNA; NC, negative control; ECM, extracellular matrix

To assess the functional role of HBEGF within chondrocytes, we next transfected these cells with HBEGF-specific overexpression (OE) or shRNA constructs. Successful HBEGF overexpression (Fig. 3A) and knockdown (Fig. 3B) in primary human chondrocytes were then confirmed by qPCR at 24 h post-transfection. Western immunoblotting was further used to confirm the overexpression or knockdown of HBEGF in appropriately transfected cells at 48 h post-transfection (Fig. 3C). MMP-3, MMP-13, and ADAMTS4 were downregulated at both the mRNA and protein levels in chondrocytes transfected with the OE HBEGF construct (Fig. 3D and E), with concomitant increases in COL2A1 and aggrecan expression. When HBEGF was silenced in chondrocytes, these cells exhibited decreases in COL2A1 and aggrecan expression together with increased MMP-3, MMP-13, and ADAMTS4 levels (Fig. 3F and G).

### HBEFG reverses the effects of miR-760 overexpression on OA progression in vitro

**Fig. 4:**
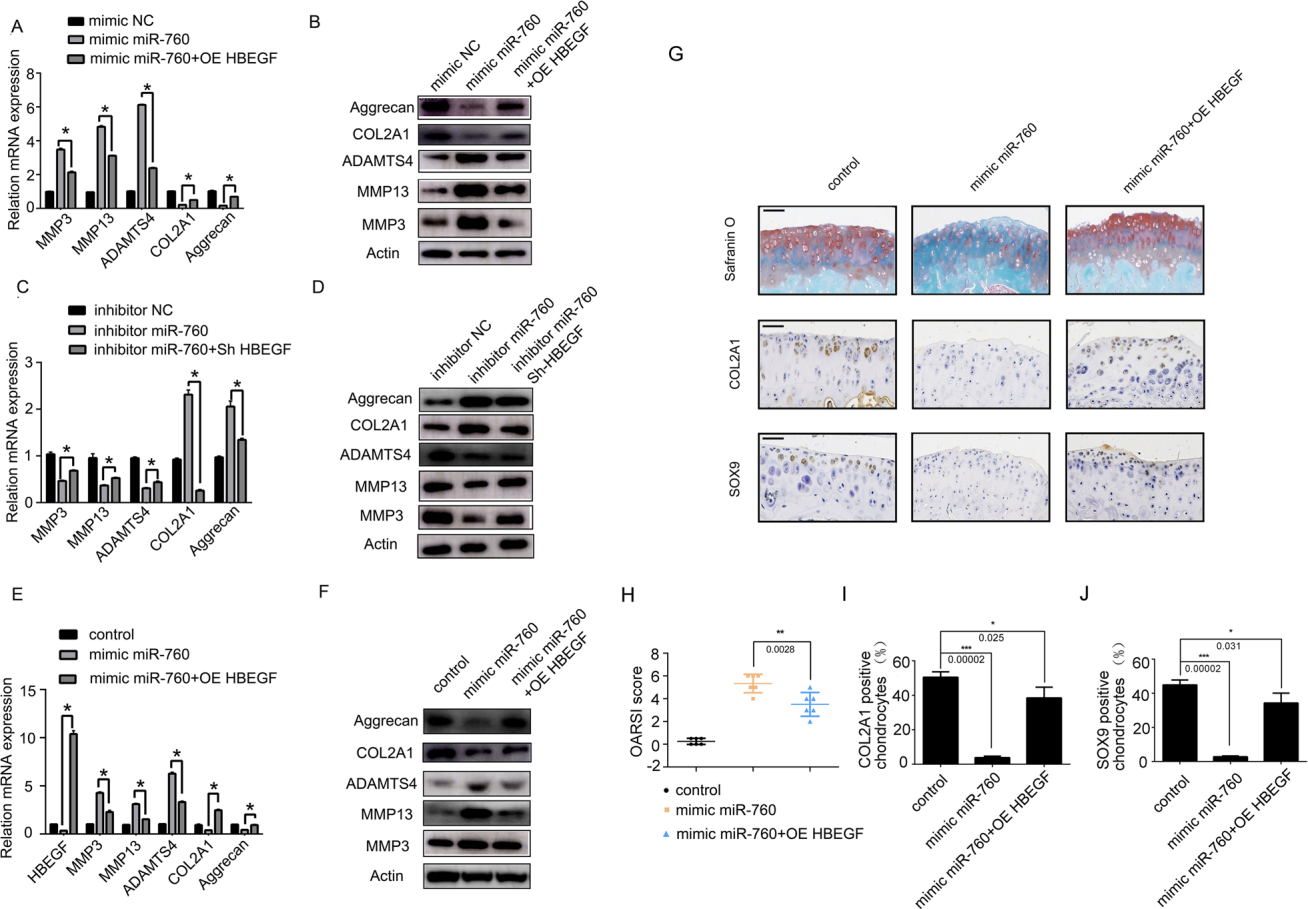
HBEGF reverses the effects of miR-760 on OA progression. **A**, **B** After 24 and 48 h of transfection, OA-associated cartilage metabolism gene expression at the mRNA (A, *n* = 3 with 3 technical replicates; data were analysed by one-way analysis of variance (ANOVA) followed by Tukey’s post hoc test for comparison between the control and treatment groups. *p < 0.05) and protein (B, *n* = 3; three different donors) levels were assessed in chondrocytes following miR-760 mimic transfection plus HBEGF overexpression. **C**, **D** OA-associated cartilage metabolism gene expression at the mRNA (C, *n* = 3 with 3 technical replicates; data were analysed by one-way analysis of variance (ANOVA) followed by Tukey’s post hoc test for comparison between the control and treatment groups. *p < 0.05) and protein (D, *n* = 3; three different donors) levels were assessed in chondrocytes following miR-760 inhibitor transfection plus HBEGF knockdown. The miR-760/HBEFG axis regulates OA progression in vivo. Sham: incision of the right knee of mice that did not undergo ACLT surgery. ACLT: right knee articular cartilage that underwent anterior cruciate ligament transection. **E**, **F** Changes in cartilage metabolism-related gene expression at the mRNA level (E, *n* = 3 with 3 technical replicates; data were analysed by one-way analysis of variance (ANOVA) followed by Tukey’s post hoc test for comparison between the control and treatment groups. *p < 0.05) and protein level (F, *n* = 3; three different animals) in the control group (Sham + PBS) and two OA experimental groups (ACLT + AVV-miR-760 mimic or ACLT + AAV-miR-760 mimic + AAV-OE HBEGF). **G** Safranin-O/fast green staining and COL2A1, SOX9 IHC staining in the control mice (Sham + PBS), and ACLT-induced OA articular cartilage (medial tibia) of mice injected with miR-760 mimic or miR-760 mimic + HBEGF overexpression. *n* = 6; six different animals. Scale bars = 50 μm. **H** OARSI scoring was performed according to staining results in the control group (Sham + PBS) and two OA experimental groups (ACLT + AVV-miR-760 mimic or ACLT + AAV-miR-760 mimic + AAV-OE HBEGF); *n* = 6; six different animals. Data were analysed by one-way analysis of variance (ANOVA) followed by Tukey’s post hoc test for comparison between the control and treatment groups. **p < 0.01. **I**, **J** Positive chondrocyte percentages of COL2A1 and SOX9 in the control group (Sham + PBS) and two OA experimental groups (ACLT + AVV-miR-760 mimic or ACLT + AAV-miR-760 mimic + AAV-OE HBEGF). Six mice were evaluated for each group, and eight sections at different sites were measured for each mouse. Data were analysed by one-way analysis of variance (ANOVA) followed by Tukey’s post hoc test for comparison between the control and treatment groups. *p < 0.05, ***p < 0.001. OA, osteoarthritis; IHC, immunohistochemistry; ACLT, anterior cruciate ligament transection; miRNAs, microRNAs; ECM, extracellular matrix. OE, overexpression; Sh, short hairpin RNA; NC, negative control

Next, primary human chondrocytes were transfected with both miR-760 mimic and HBEGF overexpression constructs. At 24 h post-transfection, MMP-3, MMP-13, ADAMTS4, aggrecan, and COL2A1 mRNA levels were assessed via qPCR, revealing that HBEGF overexpression partially reversed the effects of miR-760 overexpression on these ECM metabolism-related genes (Fig. 4A). Similarly, at 48 h post-transfection, western immunoblotting confirmed the ability of HBEGF overexpression to increase aggrecan and COL2A1 protein levels while suppressing MMP-3, MMP-13, and ADAMTS4 expression in miR-760 mimic-transfected cells (Fig. 4B). Conversely, when chondrocytes were cotransfected with miR-760 inhibitor and shHBEGF constructs, similar mRNA and protein level data were observed to those shown in Fig. 4C and D, with HBEGF knockdown thus reversing the beneficial effects of miR-760 inhibition, further confirming the identity of HBEGF as a miR-760 target gene.

### The miR-760/HB-EFG axis regulates OA progression in vivo

To extend the above in vitro findings, we assessed the ability of the miR-760/HB-EFG axis to regulate in vivo OA progression by establishing a murine ACLT-induced model of OA and providing these animals with intra-articular injections of adenoviral vectors encoding miR-760 mimic constructs with or without OE HBEGF vectors. At 8 weeks post-OA modelling, RNA and tissue samples from the knee joints of these animals were isolated for analysis, confirming the successful overexpression of HBEGF in mice in the appropriate treatment groups (Fig. 4E). qPCR and WB (Fig. 4E and F) were performed to detect the expression of catabolic enzymes (MMP-3, MMP-13 and ADAMTS4) and ECM composition (Aggrecan and COL2A1) in mouse cartilage tissues. The results showed that the injection of mimic miR-760 + OE HBEGF alleviated the degenerative changes in the cartilage matrix, such as decreased catabolic enzymes and enhanced ECM composition, in the mouse model of OA.

**Fig. 5:**
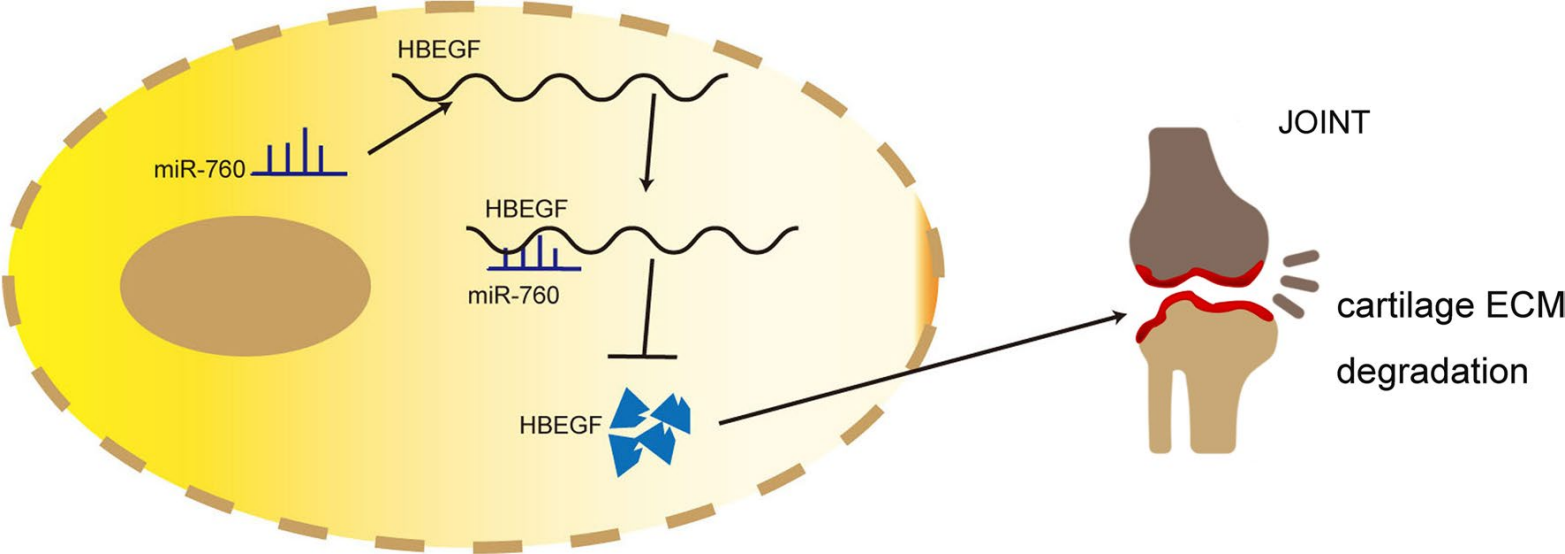
A schematic overview of the putative mechanisms whereby miR-760 regulates HBEGF expression and OA progression by influencing chondrocyte-associated ECM homeostasis

In addition, safranin O and fast green and immunohistochemistry staining (Fig. 4G) were performed. Quantitative analysis with Osteoarthritis Research Society International (OARSI) scoring showed that mimic miR-760 treatment significantly increased OARSI scores, whereas mimic miR-760 + OE HBEGF treatment lowered OARSI scores (Fig. 4H). Details about the type of OARSI scoring (mean/max/sum) are included in Additional file 2. Immunohistochemistry staining results showed that the rate of COL2A1- and SOX9-positive chondrocytes was decreased in the mimic miR-760 group, whereas the mimic miR-760 + OE HBEGF group exhibited increased ECM composition expression (Fig. 4I and J). Taken together, HBEGF overexpression partially reversed miR-760 mimic-induced exacerbation of OA severity. As such, miR-760 was able to regulate HBEGF expression and thereby drive OA progression by altering chondrocyte homeostasis and associated ECM metabolism in these animals (Fig. 5).

## Discussion

OA is a highly complex and age-related, disabling joint disease characterized by progressive loss of hyaline articular cartilage, concomitant sclerotic changes in the subchondral bone and advancement in osteophytes [3]. A range of stressful stimuli including overuse, mechanical stress, excessive loading, and joint injury can alter articular cartilage and synovial membrane tissues, driving degeneration, osteophyte development, sclerosis, and synovial inflammation that ultimately induce the progression of OA [23, 24]. Altered cartilage metabolism is a hallmark of OA onset [25], with imbalanced ECM metabolism being the primary cause of articular cartilage loss in OA patients. The maintenance of appropriate ECM homeostasis is dependent on the tightly regulated expression of catabolic and anabolic enzymes, with MMP-3, MMP-13, and MMP-9 being among the most important catabolic regulators in this setting, whereas COL2A1, proteoglycans, and aggrecan are key anabolic proteins. The present study was developed to explore the effect of miR-760 on chondrocyte-mediated maintenance of ECM homeostasis and associated OA progression.

Several miRNAs have been shown to shape the pathogenesis of OA [26, 27]. For example, miR-132 regulates PTEN/PI3K/AKT signalling activity to shape OA-related chondrocyte activity [28], while miR-126 functions by controlling MAPK signalling in a rabbit model of OA to regulate the regeneration of cartilaginous tissue. Moreover, miR-107 can target caspase-1 to influence knee articular cartilage degradation [29, 30], whereas miR-103a-3p prevents OA by targeting FGF18 [31], and the miR-296-3p/PTEN axis shapes the development of OA [32]. MiR-599 can also target Casz1 and thereby alleviate inflammation while inhibiting chondrocyte apoptosis [33]. MiR-760 targets c-Myc to suppress fat metabolism [34], in addition to controlling cellular proliferative and migratory activity by targeting the BATF3/AP-1/cyclin D1 pathway [35]. MiR-760 has previously been reported to suppress G protein-coupled receptor kinase-interacting protein 1 expression, thereby inhibiting cell growth [36]. Here, a role for miR-760 in OA development was additionally identified, and a combination of bioinformatics analyses and preliminary screening led to the identification of HBEGF as an important miR-760 target gene that was downregulated in both OA patient degenerative cartilage tissues and in chondrocytes stimulated with IL-1β/TNF-α.

HBEGF signals by binding to the epidermal growth factor receptor (EGFR), which plays important roles in tumorigenesis [37, 38], metabolic diseases [39, 40], diabetes [41], pain-related diseases [42], and Alzheimer’s disease [43]. HBEGF can modulate the pathogenesis of OA and is reportedly linked to inflammation-associated muscle injury and related regenerative activity [44]. Mice harbouring a cartilage-specific loss of EGFR expression exhibit more rapid knee OA development, whereas cartilage degeneration was suppressed in mice in which the EGFR pathway was hyperactivated, with such hyperactivation similarly reversing other OA-related pathological changes observed following surgical medial meniscus destabilization [45]. Here, HBEGF silencing was further confirmed to induce imbalanced ECM metabolic activity in chondrocytes, with OA corresponding to a reduction in HBEGF expression, whereas the overexpression of HBEGF was sufficient to suppress excessive extracellular catabolic enzyme expression in vivo and in vitro.

To our knowledge, this study is the first to examine the regulatory role of the miR-760/HBEGF signalling pathway in the context of OA. However, this study is subject to some limitations. To avoid the effects of spontaneous osteoarthritis on the joints of mice, we used a 6-week-old mouse model with a relatively normal osteochondral morphology that was relatively conducive to operation. However, the 6-week-old mouse is very young for use in an OA model, which may affect its normal bone development and repair. In addition, mimic NC (vehicle therapy) injection was missing, given that sham surgery mice injected with PBS may not rule out the effect of miRNA itself on mouse joints. Finally, as an EGFR ligand, HBEGF can bind to the EGFR receptor and thus regulate the expression of downstream signalling pathways. Consequently, the signalling pathways downstream of the miR-760/HBEGF signalling axis also remain to be identified.

## Conclusions

In summary, these experiments revealed that miR-760 is upregulated in degenerated articular tissue samples from OA patients, with the treatment of chondrocytes using inflammatory cytokines similarly driving time-dependent miR-760 upregulation. Functional analyses demonstrated the ability of miR-760 to regulate chondrocyte-mediated ECM degradation, thereby controlling the onset and progression of OA. The overexpression of miR-760 in chondrocytes was associated with significant increases in MMP3, MMP13, and ADAMTS4 expression with concomitant aggrecan and COL2A1 downregulation, whereas miR-760 inhibition had the opposite effect. Mechanistic analyses revealed that miR-760 was capable of binding the HBEGF mRNA, thereby regulating HBEGF mRNA and protein levels within chondrocytes, with HBEGF in turn controlling OA-related metabolic genes, reversing the deleterious changes in anabolic and catabolic enzyme expression induced by miR-760. In line with these in vitro results, overexpressing miR-760 in the articular cavity of OA model mice was sufficient to drive OA progression, while simultaneous miR-760 and HBEGF overexpression was associated with the loss of miR-760-driven OA progression. Together these data thus highlight the miR-760/HBEGF axis as an important mediator of OA development that may be amenable to therapeutic intervention. However, mice in which miR-760 and/or HBEGF have been knocked out will be required to guide preclinical efforts to further confirm the role of this miR-760/HBEGF axis in the regulation of OA.

## Supplementary Information

**Additional file 1.** Vector sequence diagram description.

**Additional file 2.** Supplementary table and figure.

## Data Availability

The original contributions presented in the study are included in the article, and further inquiries can be directed to the corresponding authors.

## Abbreviations

ACLT: Anterior cruciate ligament transection
DMEM: Dulbecco’s modified Eagle’s medium
EGFR: Epidermal growth factor receptor
ECM: Extracellular matrix
HBEGF: Heparin-binding EGF-like growth factor
MMP: Matrix metalloproteinase
miRNA: MicroRNA
OA: Steoarthritis
PBS: Phosphate-buffered saline
qPCR: Quantitative reverse transcriptase-polymerase chain reaction
TNF: Tumour necrosis factor
IL: Interleukin
WT: Wild type
MUT: Mutant
NC: Negative control
OE: Overexpression
Sh: Short hairpin RNA
IHC: Immunohistochemistry
AAV: Adeno-associated virus
