# Supplementary material for: MiR-760 targets HBEGF to control cartilage extracellular matrix degradation in osteoarthritis
Source: J Orthop Surg Res. 2023 Mar 10;18:186. doi: 10.1186/s13018-023-03664-1 (PMC9999495; doi:10.1186/s13018-023-03664-1)
Supplement: Supplementary file 1 — Additional file 1. Vector sequence diagram description. [file 13018_2023_3664_MOESM1_ESM.docx]

Sh HBEGF:

5’-CTAGTTTCCAAAAAGTCCGTGACTTGCAAGAGGTCTCTTGAACCTCTTGCAAGTCACGGACGGGTGCA-3’;

5’-CCCGTCCGTGACTTGCAAGAGGTTCAAGAGACCTCTTGCAAGTCACGGACTTTTTGGAAA-3’.

**Figure S1. The sequence of sh HBEGF.**

OE HBEGF:

5’-ATGAAGCTGCTGCCGTCGGTGGTGCTGAAGCTCTTTCTGGCTGCAGTTCTCTCGGCACTGGTGACTGGCGAGAGCCTGGAGCGGCTTCGGAGAGGGCTAGCTGCTGGAACCAGCAACCCGGACCCTCCCACTGTATCCACGGACCAGCTGCTACCCCTAGGAGGCGGCCGGGACCGGAAAGTCCGTGACTTGCAAGAGGCAGATCTGGACCTTTTGAGAGTCACTTTATCCTCCAAGCCACAAGCACTGGCCACACCAAACAAGGAGGAGCACGGGAAAAGAAAGAAGAAAGGCAAGGGGCTAGGGAAGAAGAGGGACCCATGTCTTCGGAAATACAAGGACTTCTGCATCCATGGAGAATGCAAATATGTGAAGGAGCTCCGGGCTCCCTCCTGCATCTGCCACCCGGGTTACCATGGAGAGAGGTGTCATGGGCTGAGCCTCCCAGTGGAAAATCGCTTATATACCTATGACCACACAACCATCCTGGCCGTGGTGGCTGTGGTGCTGTCATCTGTCTGTCTGCTGGTCATCGTGGGGCTTCTCATGTTTAGGTACCATAGGAGAGGAGGTTATGATGTGGAAAATGAAGAGAAAGTGAAGTTGGGCATGACTAATTCCCACTGA-3’.

**Figure S2. The sequence of OE HBEGF.**

5’-AAGTGGAGCAATAGCAGAGCTCGTTTAGTGACCGTCAGATCGCCTGGAGACGCCATCCACGCTGTTTTGACCTCCATAGAAGACACCGACTCTACTAGAGGATCTATTTCCGGTGAATTCACCATGTATCCATATGATGTTCCAGATTATGCTACCGGTCAATTCCTCGAGACTAGTTCTAGAGCGGCCGCGGATCCCGCCCCTCTCCCTCCCCCCCCCCTAACGTTACTGGCCGAAGCCGCTTGGAATAAGGCCGGTGTGCGTTTGTCTATATGTTATTTTCCACCATATTGCCGTCTTTTGGCAATGTGAGGGCCCGGAAACCTGGCCCTGTCTTCTTGACGAGCATTCCTAGGGGTCTTTCCCCTCTCGCCAAAGGAATGCAAGGTCTGTTGAATGTCGTGAAGGAAGCAGTTCCTCTGGAAGCTTCTTGAAGACAAACAACGTCTGTAGCGACCCTTTGCAGGCAGCGGAACCCCCCACCTGGCGACAGGTGCCTCTGCGGCCAAAAGCCACGTGTATAAGATACACCTGCAAAGGCGGCACAACCCCAGTGCCACGTTGTGAGTTGGATAGTTGTGGAAAGAGTCAAATGGCTCTCCTCAAGCGTATTCAACAAGGGGCTGAAGGATGCCCAGAAGGTACCCCATTGTATGGGATCTGATCTGGGGCCTCGGTGCACATGCTTTACATGTGTTTAGTCGAGGTTAAAAAAACGTCTAGGCCCCCCGAACCACGGGGACGTGGTTTTCCTTTGAAAAACACGATGATAAGCTTGCCACAACCCACAAGGAGACGACCTTCCATGACCGAGTACAAGCCCACGGTGCGCCTCGCCACCCGCGACGACGTCCCCCGGGCCGTACGCACCCTCGCCGCCGCGTTCGCCGACTACCCCGCCACGCGCCACACCGTCGACCCGGACCGCCACATCGAGCGGGTCACCGAGCTGCAAGAACTCTTCCTCACGCGCGTCGGGCTCGACATCGGCAAGGTGTGGGTCCCGGACAACGGCCCCCCGGTGGCGGTCTGGACCCCCCCGAAAACGTCAAAACGGGGGCGGTGTTCCCCAAATCCGCCCCCCATGGCCAAATTAACGGTTCCGGCTGGCCCCCACCACC-3’.

**Figure S3. The sequence of the corresponding negative control constructs (plvx empty vector ) of Sh HBEGF and OE HBEGF.**
