# Supplementary material for: MiR-760 targets HBEGF to control cartilage extracellular matrix degradation in osteoarthritis
Source: J Orthop Surg Res. 2023 Mar 10;18:186. doi: 10.1186/s13018-023-03664-1 (PMC9999495; doi:10.1186/s13018-023-03664-1)
Supplement: Supplementary file 2 — Additional file 2. Supplementary table and figure. [file 13018_2023_3664_MOESM2_ESM.docx]

**Table S1 Bioinformatics analysis, 188 downstream genes that are highly bound to miR-760.**


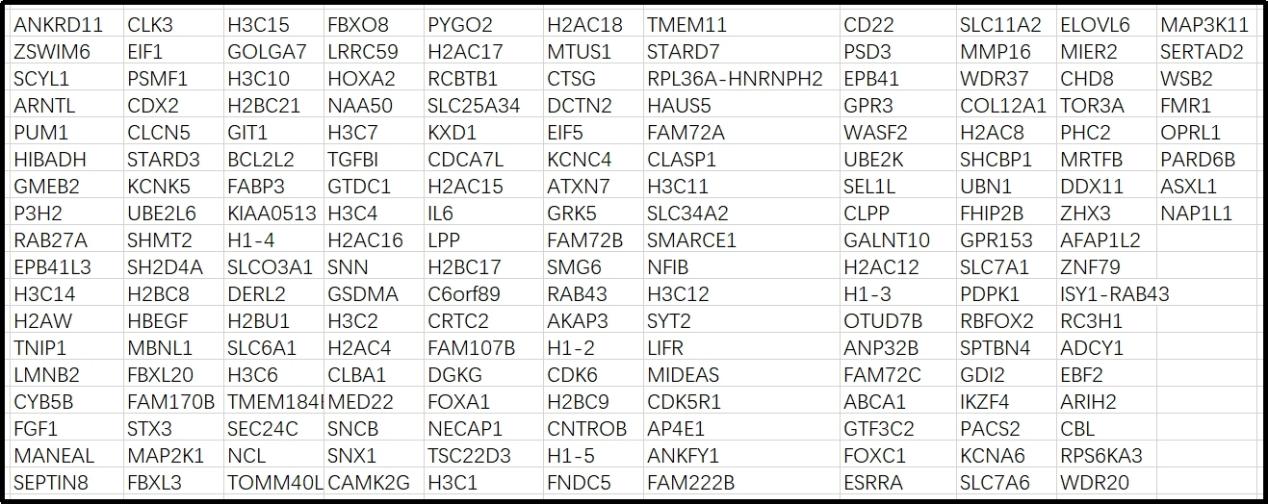


**Table S2 Bioinformatics analysis, 11 signaling pathways associated with 188 downstream genes**


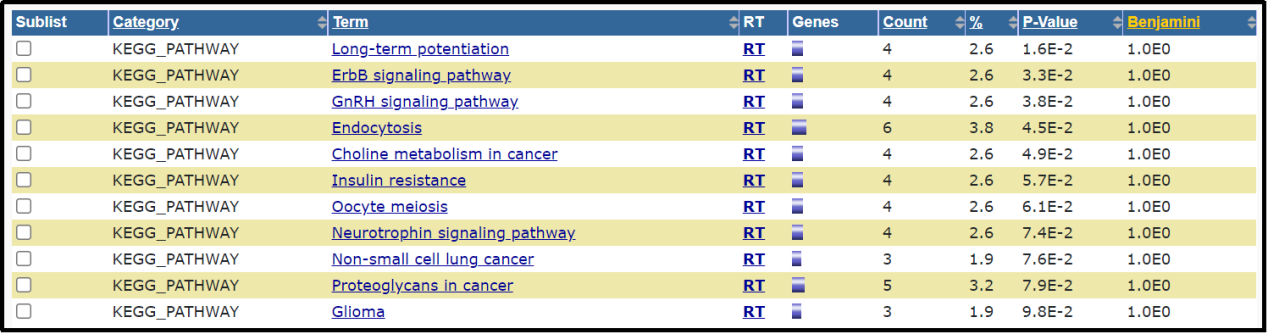


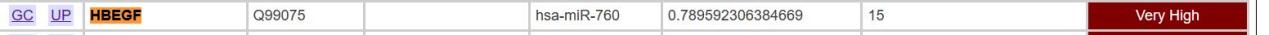


**Figure S1 HBEGF is the potential target of miR-760 with the miRDIP database and the genes with a high minimum score (top 5%).**

Position 164-170 of HBEGF 3' UTR

5' ...GCUUUGCCAAAAUACCAGAGCCU...

[hsa-miR-760](https://www.targetscan.org/cgi-bin/targetscan/vert_72/www.mirbase.org/cgi-bin/mirna_entry.pl?acc=hsa-miR-760) I I I I I I I

3'       AGGGGUGUCUGGGUCUCGGC

Position 164-170 of mutant HBEGF 3' UTR I I I I I I I

5' ...GCUUUGCCAAAAUACGAGACGGU...

**Figure S2 The details of mutant HBEGF report constructs.**

**Table S3 The details of OARSI score in three groups evaluated at week 8 after surgery. n=6.**

|  | Control | miR-760 mimic | miR-760 mimic +OE HBEGF |
| --- | --- | --- | --- |
| Mean | 0.25 | 5.33 | 3.5 |
| Max | 0.5 | 6 | 5 |
| Min | 0 | 4 | 2 |
| Sum | 1.5 | 32 | 21 |
| Stdev | 0.274 | 0.816 | 1.049 |
